# Beliefs and Perceptions of Midwives on Prevention of Mother‐to‐Child Transmission of Hepatitis B in Selected Primary Health Care Facilities in Ghana

**DOI:** 10.1155/jp/4644339

**Published:** 2026-04-08

**Authors:** Adiza Atoko Mumuni, Florence Naab, Charles Ampong Adjei, Vivian Efua Senoo-Dogbey

**Affiliations:** ^1^ Department of Maternal and Child Health, School of Nursing and Midwifery, College of Health Sciences, University of Ghana, Accra, Ghana, ug.edu.gh; ^2^ Department of Public Health, School of Nursing and Midwifery, College of Health Sciences, University of Ghana, Accra, Ghana, ug.edu.gh

## Abstract

**Background:**

Midwives play a critical role in the prevention of mother‐to‐child transmission (PMTCT) of Hepatitis B in Ghana. Their beliefs, perceptions, and attitudes may influence the uptake and delivery of preventive practices. This study used the theory of planned behavior (TPB) as a guiding framework to explore these factors.

**Method:**

A qualitative exploratory descriptive design was used to engage 14 midwives who were purposively sampled from the Madina Polyclinic, Accra, from February to April 2020. Data were collected through in‐depth semistructured interviews, transcribed verbatim, and analyzed manually using content analysis. Themes and subthemes were identified and reported.

**Result:**

The findings of the study revealed one main theme of subjective norms reflecting issues of superior influence and organization protocols, and one other main theme of attitudes, encompassing positive behaviors towards PMTCT. Midwives generally perceived hepatitis B as severe and highly infectious, adhered to protocols, and demonstrated positive engagement in vaccination, screening, and health education. Fear of infection sometimes led to exaggerated protective practices, but did not prevent care delivery.

**Conclusion:**

Midwives′ attitudes and subjective norms are important determinants of PMTCT practices. Strengthening training, supervision, and promoting hepatitis B vaccination among health care workers could reduce fear, enhance preventive practices, and contribute to HBV elimination in Ghana.

## 1. Background

Hepatitis B virus (HBV) infection remains one of the most potentially life‐threatening liver infections in the world. Evidence suggests that individuals who are exposed to HBV risk the chance of developing complications such as liver cirrhosis and liver cancer [1].

Furthermore, the focus has shifted from eliminating hepatitis B MTCT to now ensuring the triple elimination of HBV, syphilis, and HIV to ensure integrated service delivery by HCWs [2]. Global estimates show that people who are chronically infected with HBV are about 296 million (defined as hepatitis B surface antigen‐positive [HbsAg]) [1, 3]. In Africa, MTCT and horizontal routes are the major factors in the transmission of HBV, which is the main cause of chronic HBV[1, 4]. The age at which an individual develops chronic HBV infection contributes to the rate at which they develop complications. About 80%–90% of infants who are infected at birth develop chronic HBV infection later in life [1]. HBV immunoglobulin (HBIG) administration at birth, followed by HBV vaccine, is effective as postexposure prophylaxis for exposed newborns, and this has been proven to be a very useful strategy in PMTCT of HBV. Newborns have a 90% chance of being protected against HBV infection if HBIG is given correctly within 12 h after birth [1, 4, 5]. Subsequently, these babies must receive the remaining doses of the hepatitis B vaccine, which is in line with the Expanded Program on Immunization (EPI) schedule to ensure complete protection [5, 6].

Hepatitis B remains endemic in Ghana, with recent studies reporting HBsAg prevalence among pregnant women ranging from approximately 4%–10%. MTCT is a key route of infection, with recent data in Ghana indicating an MTCT rate of about 5%–6% in the absence of timely birth dose vaccination and monoprophylaxis [7–9].

Ghana has not yet implemented the universal birth dose vaccination of HBV [10]. Studies show that hepatitis B birth‐dose vaccination is not universally administered in Ghana, with significant delays and gaps in timely coverage due to vaccine availability, policy, and procedural barriers. Further, even among exposed infants, coverage is incomplete, with only about two‐thirds receiving the birth dose in some regions [7, 11].

Although policy frameworks in Ghana recommend strengthening PMTCT of hepatitis B, the birth‐dose vaccine has not been fully integrated into the EPI, and hepatitis B immunoglobulin and monovalent vaccines for exposed infants are often paid out‐of‐pocket by families[8, 12]. The routine hepatitis B vaccination schedule under the EPI still begins at 6 weeks of age [11].

Like other Sub‐Saharan African countries where MTCT of HBV contributes highly to new infections in the general population, midwives in Ghana contribute so much to the country′s efforts to eliminate HBV.

In the Ghanaian context, antenatal care is largely delivered through the focused antenatal care (FANC) model, which emphasizes early booking, targeted screening, and prevention of maternal and neonatal complications. Hepatitis B screening has increasingly been integrated into ANC services.

Midwives play a key role in the PMTCT of HBV. Their roles include providing information, education and counseling on HBV infection, its mode of transmission and prevention as part of their routine antenatal care to pregnant women. Midwives provide pretesting counseling and screening (testing women for HBsAg) to all pregnant women during their antenatal booking visits. Again, midwives are involved in posttest counseling, prescription of postexposure prophylaxis and the timely administration of HBIG to HBV‐exposed newborns. The midwives also promote follow‐up visits to assess the effectiveness of HBIG through HBsAg testing 6 months after birth and provide linkage to care for mothers and infected newborns [1, 5]. We, however, contend that midwives′ beliefs and perceptions may influence their willingness and intentions to engage in practices that are aimed at PMTCT of HBV.

## 2. Method

### 2.1. Aims and Research Design

This study is aimed at exploring midwives′ beliefs, perceptions, and attitudes towards PMTCT of hepatitis B in Ghana. A qualitative exploratory descriptive design was employed to enable participants to provide rich, detailed accounts of their experiences and practices regarding PMTCT of hepatitis B. This design was deemed appropriate to capture midwives′ subjective experiences in their natural work environment.

The study was guided by the theory of planned behavior (TPB), which posits that behaviors are influenced by attitudes, subjective norms, and perceived behavioral control. In this manuscript, we focused on subjective norms (superior influence and organizational norms) and attitudes (beliefs and perception) towards PMTCT practices, whereas the construct of perceived behavioral control and behavior has been reported in a previous publication [13]. Using the TPB allowed for a systematic examination of the cognitive and social factors shaping midwives′ PMTCT practices.

### 2.2. Study Setting

The study was conducted at Madina Polyclinic in the La Nkwantanang Municipal Area in the Greater Accra region. The study was conducted in this setting because the midwives in this setting are directly responsible for the administration of hepatitis B birth dose vaccination to babies born to mothers living with HBV. Unlike in other settings, midwives often facilitate access to the vaccine but do not administer it themselves, which highlights gaps in practice that this study aimed to capture.

### 2.3. Participants and Recruitment

Participants were registered midwives working at the Madina Polyclinic (Kekele) who provided care to pregnant women from conception to 6 weeks postpartum and had at least 1 year of working experience at the facility. Midwives whose work did not involve direct care of pregnant women, such as those working at the family planning and outreach clinics, were excluded.

A total of 14 midwives were recruited through purposive sampling, selected to provide rich and detailed insight into their practices and experiences. Data collection continued until thematic saturation was reached, which occurred after the 14th interview when no new concepts relevant to the study objectives emerged [14, 15].

### 2.4. Data Collection

Data was collected from February to April 2020 using semistructured, in‐depth interviews guided by an interview guide developed from the research objectives, TPB construct, and literature review. The interview guide included demographic questions and topics exploring midwives′ beliefs, perceptions, and attitudes towards the PMTCT of hepatitis B. The full interview guide is provided as Supporting Information.

All interviews were conducted face‐to‐face in English by the first author, with audio recording after obtaining participants′ consent. Field notes were made informally during transcription to capture nonverbal cues, such as laughter or emotional responses, which were considered in the analysis when relevant. Each interview lasted between 30 and 60 min. Rapport was established prior to each interview to encourage open discussion, and participants were reminded of their right to withdraw at any time without consequences.

### 2.5. Data Analysis

Audio‐recorded interviews were transcribed verbatim manually by the first author, and pseudonyms were assigned to ensure anonymity. Data analysis was conducted manually using content analysis as described by Kleinheksel [16]. The coding process was guided by the interview guide and the TPB framework. Codes were generated from the transcripts, grouped into subthemes, and then organized into main themes. The final coding scheme is provided as Supporting Information.

### 2.6. Rigor/Trustworthiness

In ensuring the trustworthiness of the study, the researchers ensured credibility by establishing early acquaintances and member checking to ensure that transcribed data accurately represented participants′ experiences. To ensure findings are dependable, interviews were audio recorded and data transcribed verbatim, and cross‐verified by coauthors. An audit trail documented all research decisions, including coding, theme development, and analytic processes. For transparency, field notes capturing nonverbal cues were incorporated into the analysis when relevant [16].

### 2.7. Ethics Considerations

Ethics approval was obtained from the Ghana Health Service Ethics review committee (GHS‐ERCO48/11/19). Written informed consent was obtained from each participant, and anonymity and confidentiality were maintained through pseudonyms and secure storage of all audio recordings, transcripts, and field notes. Access to the data was restricted to the research team only.

## 3. Results

### 3.1. Demographic Characteristics of Participants

A total of 14 midwives participated in the study, all female, with ages ranging from 29 to 50 years (mean 34). Most participants held a diploma in midwifery, whereas a few had a bachelor′s degree in nursing or midwifery. Their years of professional experience ranged from 3 to 14 years, and participants held ranks from midwifery officer to senior midwifery officer.

Two themes and five subthemes emerged from this study. The themes and subthemes are summarized in Table 1.

**Table 1:** Categories of beliefs and perceptions.

Major themes	Subthemes
Attitude of midwives towards hepatitis B and PMTCT	a) Knowledge, perception, and beliefs about hepatitis B
	b) Positive attitudes towards PMTCT of hepatitis B
	c) Fear‐driven and cautious attitudes towards PMTCT
	d) Paying more attention to HIV than hepatitis B
Subjective norms influencing PMTCT of hepatitis B	e) Influence of superiors and institutional protocols

### 3.2. Theme 1: Attitude of Midwives Towards Hepatitis B and PMTCT

Based on the TPB, midwives′ attitudes towards hepatitis B and its prevention were shaped by their knowledge, perception, and beliefs about the disease, as well as their experiences in clinical practice. These attitudes influenced how midwives perceived the risk of infection and how they cared for pregnant women living with hepatitis B.

### 3.3. Knowledge, Perception, and Beliefs About Hepatitis B

Participants described different perceptions about hepatitis B before receiving professional training. Most participants perceived hepatitis B as a medical condition and did not associate it with spiritual or cultural causes. However, one participant reported that before becoming a midwife, she believed hepatitis B had a spiritual cause.

*Before I got to study about it, I was thinking it was one of those spiritual diseases that people get. Especially when you see them with swollen legs and feet, but when I came into the world of health and studied, I saw that it isn′t a spiritual something but a condition that can also be prevented **(Gifty).***

Other participants strongly disagreed with spiritual explanations and emphasized that hepatitis B is a biomedical condition.

*I think it′s just like any sickness. I don′t see it as spiritual, my opinion says, since it has been detected, it cannot be spiritual. Any spiritual illness in our beliefs will not be detected in the hospital, so I believe if you come and it is tested, then you will get the opportunity to go through the management process **(Precious).***

Similarly, Katako stated:

*I don′t think it′s a spiritual disease. I have never attributed any form of sickness to a spiritual cause before **(Katako).***

Most participants believed that hepatitis B could be transmitted through saliva and poor hygiene.

*If I have it in my saliva and I spit, and mistakenly it touches you, and you don′t wash your hands well, and you use it on any part of your body, you can also acquire it. So, I think it is contagious **(Gifty).***

Another participant believed that hepatitis B could be transmitted through sweat.

*Yeah, it is very contagious. I learnt you can even get it through sweat when a person sweats, and you touch the person, you can even get it, yeah **(Ortin).***

Participants also expressed beliefs about the severity of hepatitis B.

*I see it to be severe because if someone is Hepatitis B positive and I have a cut and the person′s blood gets into my bloodstream, I don′t think It will spare me. I will get it so I can see if it is severe. Even when you share the same cup with someone who has it you will get it; that is the only thing I can say **(Boosua).***

However, one participant expressed a different view.

*I wouldn′t say it is severe because it doesn′t deteriorate the person′s health very fast, so I would not say that. Even though we know the long-term effect can be fatal (**Anastasia**).*

Participants also believed that hepatitis B is highly infectious.

*When taking care of a very ill, infected Hepatitis B patient, the body fluids alone that you will be in contact with can make you acquire the infection. I have an example of a friend like that. She said a friend of hers was very sick, she took care of her, and later the friend died, and they told her she died of Hepatitis B. Since then, she has tested positive for Hepatitis B (**Agnes).***

### 3.4. Positive Attitude Towards PMTCT of Hepatitis B

Most participants expressed positive attitudes towards the PMTCT of hepatitis B. They believed PMTCT was important in ensuring the health of both mother and baby and in preventing avoidable illness in newborns.

*PMTCT is essential because we can’t let the baby have it. Once you can prevent it, why not? Why will you infect your baby with Hepatitis B, which can be chronic and can cause the death of the baby, just like any other STI/HIV, so it is very essential **(Katako).***

Similarly, Precious stated:

*It is every midwife′s dream and every midwife′s wish that when she sees a pregnant woman, the woman will deliver safely, and then the baby will be free from any preventable diseases. And I want her to deliver safely and the baby to be healthy so that we will have under-fives been healthy and then growing into healthy adults **(Precious).***

Some participants also emphasized the importance of nonstigmatizing care.

*Where I work, it is normally like we have been educated against stigmatization, it is just like HIV, they say we should not stigmatise. All that you have to do is to protect yourself well and then give the necessary care that you are supposed to give. **(Davi).***

### 3.5. Fear‐Driven and Cautious Attitudes Towards PMTCT

Despite attitudes, some participants described fear‐driven behaviors when caring for pregnant women living with hepatitis B. These behaviors included excessive use of protective clothing and heightened caution.

*Some of us exaggerate when it comes to wearing protective clothing. Some can wear three gloves, yes; I have seen one before. You know, normally we don’t usually wear the boot, but that day the person put on the boot, apron and wore about five (5) gloves **(Ortin).***

Kakra shared her experience as a newly posted midwife

*When you come out of school like that with all the knowledge, and you come to the field, and you see somebody with Hepatitis B, you will be like, I must treat her just like an HIV patient. I had to wear double gloves each time I am attending to the client; I must be very cautious, but now, even though I am cautious, I am ok psychologically **(Kakra).***

Precious also observed similar behaviors among colleagues.

*We are humans, and no matter how protected you are, you are very cautious of things like that, so yes, I have seen midwives who are cautious, putting on extra gloves, telling other colleagues to be careful because she is this and that. **(Precious)***

Agnes added:

*Interestingly, there are some cases when you start handling them immediately, you will be prompted to be careful, she is this, she is that, it happens because sometimes we are scared **(Agnes).***

### 3.6. Paying Attention to HIV Rather Than Hepatitis B

Some participants reported that hepatitis B was not given the same level of attention as HIV during labor and delivery. According to them, HIV status was checked and acted upon more quickly than hepatitis B.

*When the person comes in labour, the first thing they check is HIV, not hepatitis B. So mostly we can take care of the woman till like 24 hours before we realise that she is Hepatitis B positive because HIV is what everybody is looking at. **(Katako)***

Another participant shared a similar observation.

*Hepatitis B is a condition that people don’t fear as much as HIV, so we see it as normal compared to a client who has HIV **(Patri).***

### 3.7. Theme 2: Subjective Norms Influencing PMTCT of Hepatitis B

Using the TPB, these subjective norms reflected the perceived social pressures on midwives, including the influence of superiors and organizational protocols, which shaped their engagement in PMTCT practices.

Midwives described that their practices related to PMTCT were mainly guided by protocols and professional expectations rather than direct pressure from superiors.

### 3.8. Influence of Superior and Institutional Protocols

Participants indicated that protocols guided their work; failure to follow them could result in questioning.

*Generally, our work is based on protocols, so nobody will compel you to do what you must do **(Kira).***

She further explained:

*You will be questioned about it because it is something you must do; there is a protocol you must follow. So, per the outcome of whatever you are doing, and it is realised that you are not putting in much effort to combat whatever you have, you will be questioned about it **(Kira).***

Nana Yaa also emphasized the role of protocols.

*We have protocols when it comes to Hepatitis B clients, like clients who are positive. We have protocols that we follow in order for them to come out safely themselves and their baby **(Nana Yaa).***

However, some participants reported limited supervision and a lack of follow‐up.

*No, I don′t get any pressure from anybody; nobody will penalise me, but as a health worker, you know that is what you must do, so negligence is wrong, meaning your conscience will not serve you right **(Kakra).***

Another participant noted:

*There is no book for us to record in whenever we give the vaccine, so we don′t record it. If it is something that they will penalise you for, there should be a book for it so documentation will be done that so and so also came with Hep B and that after delivery, this has been done. So, I will not be penalised. They don′t follow up **(Boosua).***

## 4. Discussion

The current study explored midwives′ attitudes, perceptions and subjective norms regarding hepatitis B and their influence on practices related to PMTCT. The study established that the beliefs and perceptions about hepatitis B shaped midwives′ attitudes and behaviors in caring for pregnant women living with hepatitis B infection.

### 4.1. Beliefs and Perceptions About Hepatitis B

Midwives in this study generally perceived hepatitis B as a contagious and potentially severe infection. Many participants described beliefs that causal contact and exposure to body fluids could lead to infection, which may partly explain the heightened caution seen in clinical practice. Other studies have noted that fear of infection influences healthcare workers′ behaviors towards people living with hepatitis B (PLWHB) [1, 17].

In addition, participants in this study did not attribute hepatitis B to supernatural causes, contrasting with previous research in Ghana where spiritual explanations were common [11, 18]. This difference may be due to participants′ professional exposure and access to biomedical information prior to and during training. This is mainly because the study participants are not patients living with hepatitis B and hence have a different orientation.

### 4.2. Subjective Norms and Institutional Influence

Midwives reported that their practices were guided primarily by institutional protocols rather than direct influence from superiors. Although midwives′ practices are guided by protocols and the norms laid down by their superiors, it is interesting to note that these are influential factors in their practices, as similarly reported in the literature [19, 20]. However, participants noted little supervision or follow‐up to ensure consistent application of these PMTCT guidelines. Meanwhile, existing literature has emphasized the importance of clinical supervision by superiors on clinical practice to improve the quality of health services provided, prevent burnout, increase professional competence, and provide the opportunity to evaluate clinical and cultural practices [21]. Also, a study by Gera et al. highlighted the importance of supportive supervision on service delivery [22]. The lack of structured monitoring or documentation mechanisms may reduce accountability and weaken the implementation of PMTCT practices.

### 4.3. Attitudes Towards PMTCT

Positive attitudes were reflected in participants′ willingness to provide care and follow preventive practices such as screening, health education, and vaccination. Many midwives expressed a sense of professional responsibility and felt protected by standard precautions when caring for women living with hepatitis B. Similar positive attitudes have been reported in other settings where health care workers′ knowledge of hepatitis B complications motivated engagement in preventive practices [23, 24]. These seem to suggest that a positive attitude, such as willingness to care for pregnant women living with hepatitis B by midwives, may encourage pregnant women to feel safe, attend antenatal clinic, and reduce MTCT of hepatitis B.

However, some participants expressed fear‐driven and overly cautious behaviors, such as excessive use of protective clothing and alerting colleagues about clients′ infection status. These findings were reported in Ghana previously, where participants were excessively cautious and breached confidentiality when caring for people with hepatitis [11]. Likewise, Scheunin in India reported healthcare workers manifested as excessive cautiousness during procedures such as blood transfusion and sample taking [23]. Although these behaviors did not result in refusal of care or postpone their duties, they engaged in task shifting or hesitated when rendering care to pregnant women with hepatitis B, as reported previously [17, 18]. Their behaviors could be interpreted by pregnant women as discriminatory, potentially affecting trust and future engagement with antenatal services.

### 4.4. Contextualizing Fear Among Healthcare Workers

Several Studies have reported that healthcare workers are more susceptible to HBV infection than the general population [1, 25, 26]. A study among HCWs in Southern Ghana found an HBsAg prevalence of 5.9% (95% CI: 3.0–8.0), indicating intermediate endemicity in this occupational group. This prevalence is like pooled estimates for Africa (approximately 6.8%) but lower than reported rates in the general Ghanaian population [27, 28]. Suboptimal uptake of hepatitis B vaccination, postexposure prophylaxis, and standard precaution among HCWs has been documented, suggesting suboptimal protection despite occupational risk [29]. Although hepatitis B vaccination is recommended for HCWs in Ghana, coverage is not universal [30]. This could be the reason why midwives in this current study expressed heightened fear and excessive cautiousness behaviors. These behaviors were also consistent with the findings by Almutairi 2017 in Saudi Arabia [22, 31].

### 4.5. Implication for Practice

The findings suggest that targeted in‐service training and supportive supervision are essential to strengthen midwives′ engagement in PMTCT practices for hepatitis B. Promoting hepatitis B vaccination among HCWs may reduce fear‐driven behaviors and enhance confidence in caring for pregnant women living with HBV. Strengthening these measures could improve the quality of care and contribute to achieving national HBV elimination.

### 4.6. Limitation

This study was conducted at a single healthcare facility, which may limit generalization to other regions of Ghana. Only midwives who were available and willing to participate were included, introducing possible selection bias. Additionally, self‐reported attitudes and behaviors may be influenced by social desirability. Furthermore, although PMTCT of hepatitis B is inherently multidisciplinary, this study focused exclusively on midwives to enable an in‐depth exploration of their experiences as a key cadre directly involved in antenatal screening, counseling, and administration of the hepatitis B birth‐dose vaccine. However, the exclusion of other stakeholders, such as physicians and nurses, may limit a broader understanding of barriers and facilitators to PMTCT implementation.

## 5. Conclusion

This study highlights that midwives′ beliefs, perceptions, and attitudes influence their engagement in PMTCT practices for hepatitis B. Although most midwives demonstrated positive attitudes towards care, fear‐driven behaviors remain a concern. Targeted training, supportive supervision, and promoting hepatitis B vaccination among HCWs are essential to reduce fear, improve quality of care, and support the elimination of HBV in Ghana.

## Author Contributions

A.A.M.: Conceptualization of the study. The manuscript writing was done by A.A.M., F.N., C.A.A. and V.E.S‐D.

## Funding

No funding was received for this manuscript.

## Disclosure

All the authors read the final manuscript. This manuscript is derived from a thesis submitted to the University of Ghana and is available on the UGSPACE [32].

## Ethics Statement

Ethics approval was obtained from the Ghana Health Service Ethics review committee (GHS‐ERCO48/11/19). Informed consent was obtained from each participant with an assurance of anonymity and confidentiality. The right to withdraw from participating in the study at any point was allowed with no consequences. Anonymity was maintained by assigning pseudonyms to participants, and confidentiality was also maintained by making sure all audio tapes, transcribed data, field notes, and documented information given by the participants were stored and the data was encrypted. Access to the data was restricted to the research team alone. We confirm that all methods were carried out in accordance with relevant guidelines and regulations.

## Consent

The authors have nothing to report.

## Conflicts of Interest

The authors declare no conflicts of interest.

## Supporting information


**Supporting Information** Additional supporting information can be found online in the Supporting Information section. Supplementary Material 1 Interview guide used for data collection.Supplementary Material 2 Final Coding Scheme developed during thematic analysis.

## Data Availability

The data that support the findings of this study are available from the corresponding author upon reasonable request.
