# Supplementary material for: Beliefs and Perceptions of Midwives on Prevention of Mother‐to‐Child Transmission of Hepatitis B in Selected Primary Health Care Facilities in Ghana
Source: J Pregnancy. 2026 Apr 8;2026:4644339. doi: 10.1155/jp/4644339 (PMC13058810; doi:10.1155/jp/4644339)
Supplement: Supplementary file 1 — Supporting Information Additional supporting information can be found online in the Supporting Information section. Supplementary Material 1 Interview guide used for data collection.Supplementary Material 2 Final Coding Scheme developed during thematic analysis. [file JP-2026-4644339-s001.docx]

**Supplementary Table 1: Final Coding Scheme.**

| **Theme** | **Sub-theme** | **Description/Example quotes** |
| --- | --- | --- |
| **Attitudes towards PMTCT** | 1. Knowledge, perception and beliefs about Hepatitis B | Beliefs about severity and contagiousness of Hepatits B and views on causes of Hepatitis B prior to professional training  *“Before I got to study about it, I was thinking it was one of those spiritual diseases that people get. Especially when you see them with swollen legs and feet, but when I came into the world of health and studied, I saw that it isn’t a spiritual something but a condition that can also be prevented”* ***(Gifty).***  *“I think is just like any sickness. I don’t see it as spiritual my opinion says since it has been detected it cannot be spiritual. Any spiritual illness in our beliefs will not be detected in the hospital so I believe if you came and it is tested then you will get the opportunity to go through the management process”* ***(Precious).***  *“I don’t think is a spiritual disease. I have never attributed any form sickness to a spiritual cause before”* ***(Katako).***  *“If I have it in my saliva and I spit and mistakenly it touches you and you don’t wash your hands well and you use it on any part of your body, you can also acquire it. So, I think it is contagious”* ***(Gifty).***  *“Yeah, it is very contagious I learnt you can even get it through sweat when a person sweats and you touch the person you can even get it yeah”* ***(Ortin).***  *“I see it to be severe because if someone is Hepatitis B positive and I have a cut and the person’s blood gets into my blood stream I don’t think It will spare me. I will get it so I see it to be severe. Even when you share the same cup with someone who has it you will get it that is the only thing I can say”* ***(Boosua).***  *“I wouldn’t say it is severe because it doesn’t deteriorate the person’s health very fast so I would not say that. Even though we know the long-term effect can be fatal “(****Anastasia****).*  *“When taking care of a very ill infected Hepatitis B patient the body fluids alone that you will be in contact with can make you acquire the infection. I have an example of a friend like that, she said a friend of hers was very sick she took care of her and later the friend died and they told her she died of Hepatitis B, since then she has tested positive for Hepatitis B” (****Agnes).*** |
|  | 1. Positive attitudes towards PMTCT of Hepatitis B | Midwives’ actions and attitudes while caring for pregnant women with Hepatitis B, Including willingness to care for positive women  *“PMTCT is very essential because we can’t let the baby have it, once you can prevent it why not? Why will you infect your baby with Hepatitis B which can be chronic and can cause the death of the baby just like any other STI/HIV so is very essential”* ***(Katako).***  *“it is every midwife’s dream and every midwife’s wish that when she sees a pregnant woman the woman will deliver safely and then baby will be free from any preventable diseases. And I want her to deliver safely and baby to be healthy so that we will have under-fives been healthy and then growing into a healthy adult”* ***(Precious).***  *“Where I work normally, it is like we have been educated against stigmatization, it is just like the HIV, they say we should not stigmatize. All that you have to do is to protect yourself well and then give the necessary care that you are supposed to give”.* ***(Davi).*** |
|  | 1. Fear-driven and cautious attitudes towards PMTCT | Fear-driven behaviour or over contiousness of midwives when providing care to women with living with Hepatitis B  *“Some of us are exaggerate when it comes to wearing protective clothing. Some can wear three gloves yes; I have seen one before.* *You know normally we don’t usually wear the boot but that day the person put on the boot, apron and wore about five (5) gloves”* ***(Ortin).***  *“When you come out of school like that with all the knowledge and you come to the field and you see somebody with Hepatitis B, you will be like, I must treat her just like HIV patient. I had to wear double gloves each time I am attending to the client; I must be very cautious but now even though I am cautious, I am ok psychologically”* ***(Kakra).***  *“We are humans and no matter how protected you are you are very cautious of things like that, so yes, I have seen midwives who are cautious, putting on extra gloves, telling other colleagues to be careful because she is this and that”.* ***(Precious)***  *“Interestingly, there are some cases when you start handling them immediately you will be prompted to be careful, she is this, she is that it happens because sometimes we are scared”* ***(Agnes).*** |
|  | 1. Paying more attention to HIV than Hepatitis B | Paying more attention to women who are HIV positive.  *“When the person comes in labour the first thing they check is the HIV not hepatitis B. So mostly we can take care of the woman till like 24hours before we realize that she is Hepatitis B positive because HIV is what everybody is looking at”.* ***(Katako)***  *“Hepatitis B is a condition that people don’t fear as much as HIV, so we see it to be normal compared to a client that has HIV “****(Patri).*** |
| **Subjective norms influencing PMTCT of Hepatitis B** | 1. Influence of superior and institutional protocols | Influence of superiors and protocols on midwives PMTCT practices.  *“Generally, our work is based on protocols so no nobody will compel you to do what you must do* ***(Kira).***  *You will be questioned about it because is it something you must do, there is a protocol you must follow. So, per the outcome of whatever you are doing, and it is realized that you are not putting in much effort to combat whatever you have to you will be questioned about it “****(Kira).***  *“We have protocols when it comes to Hepatitis B clients, like clients who are positive. We have protocols that we follow in other for them to come out safely themselves and their baby”* ***(Nana Yaa).***  “*No, I don’t get any pressure from anybody, nobody will penalize me but as a health worker you know that is what you must do so negligence is wrong meaning your conscience will not serve you right”* ***(Kakra).***  *“there is no book for us to record in whenever we give the vaccine, so we don’t record it. If it is something that they will penalize you for, there should be a book for it so documentation will be done that so so and so also came with Hep B and that after delivery this has been done. So, I will not be penalized. They don’t follow up”* ***(Boosua).*** |
